# Comparison of Fracture Resistance and Microleakage Properties of Two Different Prefabricated Zirconia Crowns After Thermocycling: An In Vitro Study

**DOI:** 10.3390/biomimetics10080538

**Published:** 2025-08-16

**Authors:** Nazile Pehlivan, Nurhan Öztaş Kırmızı, Menekşe Alim

**Affiliations:** 1 Şehit Mehmet Kılınç Oral and Dental Health Hospital, Yeşilyurt, Malatya 44900, Turkey; 2 Department of Pediatric Dentistry, Faculty of Dentistry, Gazi University, Çankaya, Ankara 06690, Turkey

**Keywords:** prefabricated zirconia crowns, fracture resistance, microleakage, pediatric dentistry

## Abstract

Biomimetic restorative treatments in pediatric dentistry increase the longevity of the restoration compared to traditional methods and aim to preserve the natural tooth structure. Prefabricated zirconia crowns have been developed as aesthetic alternatives to stainless steel crowns for full-coronal restorations of primary teeth. This study aimed to compare the fracture resistance and microleakage of two different posterior zirconia crown brands—NuSmile^®^ (USA) and ProfZrCrown^®^ (Turkey)—cemented with either conventional glass ionomer cement (GIC) or resin-modified glass ionomer cement (RMGIC). Eighty extracted primary molars were divided into four groups (n = 20). Crowns were cemented with Ketac™ Cem Radiopaque (GIC) or Ketac™ Cem Plus (RMGIC), in accordance with the manufacturers’ instructions, and then subjected to thermocycling. Fracture resistance was tested on 40 samples by applying an increasing compressive load until failure, with values recorded in Newtons (N). The remaining 40 samples were immersed in basic fuchsin dye for microleakage testing and evaluated under a stereomicroscope at 30× magnification. The results revealed that the ProfZrCrown^®^/RMGIC group exhibited significantly higher fracture resistance compared to the NuSmile^®^/RMGIC group (*p* < 0.05). No statistically significant differences were found among the other groups. Although no significant differences in microleakage were observed among the groups (*p* > 0.05), crowns cemented with GIC demonstrated higher microleakage levels. Within the limitations of this in vitro study, ProfZrCrown^®^ may be considered a promising alternative for aesthetic posterior restorations in pediatric dentistry.

## 1. Introduction

Dental caries is a widespread public health issue, particularly in developing countries, where it affects 60–90% of children [1]. The healthy preservation of primary teeth is of great importance for the overall growth and development of children, as primary teeth perform vital functions, such as mastication, aesthetics, phonetics, and guiding the eruption of permanent teeth. Due to their structural differences, caries progression in primary teeth occurs more rapidly compared to permanent teeth, and their morphological characteristics often adversely affect the success of restorations [2].

Conventional restorative approaches using resin-based materials in primary molars with extensive tissue loss frequently result in microleakage and mechanical failure [3]. In contrast, crown restorations are considered a more appropriate treatment approach for primary teeth with significant structural loss, due to their advantages, such as preservation of the mesiodistal dimension, maintenance of structural integrity, biocompatibility, and prolonged functional lifespan of the tooth [4]. The American Academy of Pediatric Dentistry (AAPD) recommends the use of stainless steel crowns (SSCs) for the restoration of extensively carious primary anterior and posterior teeth due to their superior long-term durability compared to other restorative options [5]. However, the inadequate aesthetics of stainless steel crowns (SSCs) have led to the development of alternative full-coverage restorative options [6,7,8]. Resin composite strip crowns (SCs) and prefabricated crowns, such as preveneered stainless steel crowns (PVSSCs) and prefabricated zirconia crowns (PZCs), are the options for aesthetic full-coverage restorations [9]. Each crown type has its own advantages and disadvantages. Walia et al. reported that SCs had lower retention (78%), mainly due to moisture contamination. PVSSCs had better retention (95%), but dislodgement of facial veneers was a problem, and both crowns led to increased gingival inflammation [10]. Primary anterior PZCs are a more recent addition to prefabricated pediatric crowns with promising proportions of retention as well as patient and dentist satisfaction [9,11]. An attractive alternative with good mechanical qualities, high compressive strength (2000 MPa), high flexural strength (900–1200 MPa), and fracture toughness of 6–8 MPa is zirconium, a polycrystalline ceramic devoid of glass components [12]. The toughness of zirconium materials is based on martensitic transformation. To increase the stability and thermophysical properties of ZrO_2_, stabilizers such as calcium oxide (CaO), magnesium oxide (MgO), yttrium oxide (Y_2_O_3_), and cerium oxide (CeO_2_) are often added [13]. Yttrium oxide increases the chemical and physical stability of zirconia, has low thermal conductivity, and has superior corrosion resistance [14]. Differences in the material composition of zirconium crowns can affect their fracture resistance. This study evaluated the fracture resistance properties of ProfZrCrown^®^ brand zirconium crowns, a product not yet known in the literature, and aimed to provide a basis for future studies.

Prefabricated crowns are prone to microleakage due to their open margins; therefore, the primary responsibility for minimizing microleakage lies with the luting cement. Historically, zinc phosphate and polycarboxylate cements have been used for crown cementation [15]. However, glass ionomer cements (GICs) and resin-modified glass ionomer cements (RMGICs) have demonstrated reduced coronal microleakage and lower rates of clinical failure [16].

The aim of this in vitro study was to compare the degree of microleakage associated with GIC and RMGIC cementation and to evaluate whether different brands of zirconia crowns show differences in microleakage and fracture resistance following thermocycling.

## 2. Materials and Methods

This in vitro experimental study was conducted at the Department of Pediatric Dentistry, Faculty of Dentistry, Gazi University, and the laboratory procedures were performed at the Ankara University Research Laboratory. Ethical approval was obtained from the Clinical Research Ethics Committee of Gazi University Faculty of Dentistry (Decision No: E-21071282-050.99-535439).

### 2.1. Sample Size Determination and Tooth Allocation

Based on a power analysis conducted using G*Power 3.1.9.2 software (α = 0.05, effect size = 0.5884, power = 0.80), the minimum required sample size was determined to be 9 specimens per group.

A total of 80 extracted human primary molars, free of caries or with enamel-level caries and less than one-third root resorption, were included. The teeth were randomly divided into four groups (n = 20 per group):

- Group 1: NuSmile^®^ + GIC (Ketac™ Cem Radiopaque);
- Group 2: ProfZrCrown^®^ + GIC;
- Group 3: NuSmile^®^ + RMGIC (Ketac™ Cem Plus);
- Group 4: ProfZrCrown^®^ + RMGIC.

Each group was further divided into two subgroups:

- 10 teeth for fracture resistance testing;
- 10 teeth for microleakage testing.

The material composition of two brands of prefabricated zirconia crowns is presented in Table 1.

### 2.2. Sample Preparation

The teeth were cleaned and disinfected by immersion in a 0.1% thymol solution for 24 h. Following disinfection, the teeth were stored in distilled water throughout the study period, with the water being replaced weekly. For the fracture resistance test, 40 extracted primary molars were embedded in appropriately sized acrylic blocks using cold-cure acrylic, ensuring that the cementoenamel junction remained above the acrylic surface and that the long axis of each tooth was positioned perpendicular to the base. After selecting the appropriately sized prefabricated zirconia crown, tooth preparation was performed by the same investigator in accordance with the manufacturer’s instructions. For the microleakage test, the preparation of the 40 primary molars was completed manually before embedding them in acrylic blocks. The preparation process was finalized after confirming that the crowns fit passively.

Before cementation, the prepared tooth surfaces were cleaned with pumice and then rinsed with water and dried with air. No surface treatment was applied to the internal surface of the zirconia crowns. The crowns were cemented according to the manufacturer’s instructions. All samples were thermocycled (SD Mechatronik Thermocycler, Feldkirchen-Westerham, Germany) between 5 °C and 55 °C for 6.000 cycles to simulate oral temperature variations.

### 2.3. Fracture Resistance Testing

**Figure 1:**
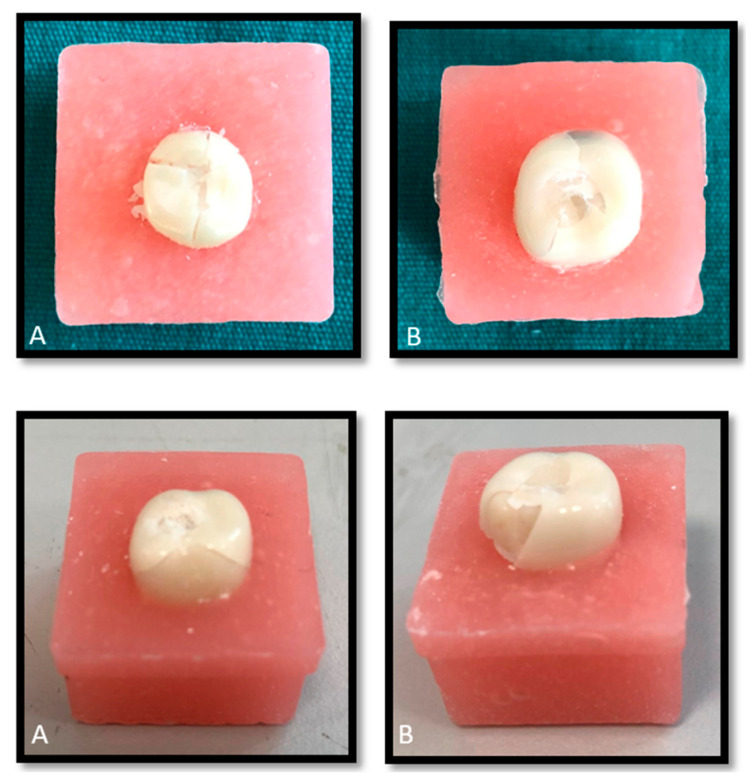
Examples of fractured specimens. (**A**): NuSmile^®^; (**B**): ProfZrCrown^®^.

The fracture resistance test was performed on 40 extracted primary molar teeth embedded in acrylic resin blocks. A progressively increasing load was applied to the center of the occlusal surface of each specimen using a 5 mm diameter stainless steel ball at a crosshead speed of 1 mm/min, perpendicular to the occlusal plane. The loading was continued until the occurrence of the first visible structural fracture, and the values were recorded in Newtons (N) using the integrated software of the testing machine (Lloyd LRX, Fareham, UK). Figure 1 illustrates some examples of fractured specimens.

### 2.4. Microleakage Testing

In the 40 specimens subjected to thermal cycling, all surfaces located 1 mm below the restoration margins were coated with two layers of nail varnish for isolation. The apical foramen was sealed with wax to prevent dye penetration. The specimens were then immersed in a 2% basic fuchsin solution (Beslab, Ankara, Turkey) for 24 h, rinsed under running water, and embedded vertically in cold-cure acrylic resin blocks, ensuring that the cemento-enamel junction remained exposed.

**Figure 2:**
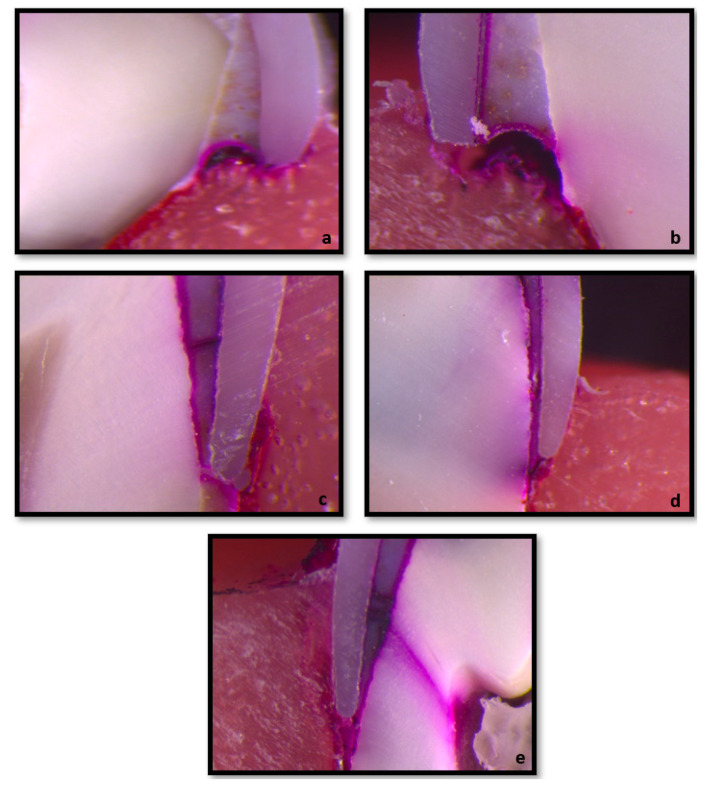
Some representative sectional images obtained from the samples are as follows: (**a**) RMGIC/ProfZrCrown^®^ microleakage score 0; (**b**) GIC/ProfZrCrown^®^ microleakage score 1; (**c**) RMGIC/ProfZrCrown^®^ microleakage score 2; (**d**) RMGIC/NuSmile^®^ microleakage score 3; (**e**) GIC/NuSmile^®^ microleakage score 4.

Buccolingual sections were obtained from the prepared specimens using a precision cutting device (Micracut 201, Metkon Instruments Ltd., Bursa, Turkey). The sections were examined under a stereomicroscope (Leica MZ12, Germany) at 30× magnification, and microleakage was evaluated according to the scoring system (Table 2):

Representative sectional images obtained from the samples are presented in Figure 2.

Intra-operator reliability testing was conducted by comparing measurements taken one week apart on 20 samples. The Kappa value obtained was 0.89, indicating almost perfect consistency between repeated measurements by the same rater.

### 2.5. Statistical Analysis

Data were analyzed using IBM SPSS Statistics v25. The Shapiro–Wilk test was used to assess the normality of the data. For comparisons among three or more groups that did not exhibit normal distribution, the Kruskal–Wallis test was applied, followed by post hoc tests with Bonferroni correction to identify the specific group or groups responsible for the difference. Fisher’s exact test was used for categorical variables. A *p*-value of < 0.05 was considered statistically significant.

## 3. Results

### 3.1. Fracture Resistance Test Results

Table 3 presents the mean and standard deviation values of the groups tested in this study. A statistically significant difference was found between the NuSmile^®^/RMGIC and ProfZrCrown^®^/RMGIC groups (*p* = 0.012), with ProfZrCrown^®^/RMGIC showing higher fracture resistance. No significant differences were observed among the other groups (*p* > 0.05).

**Figure 3:**
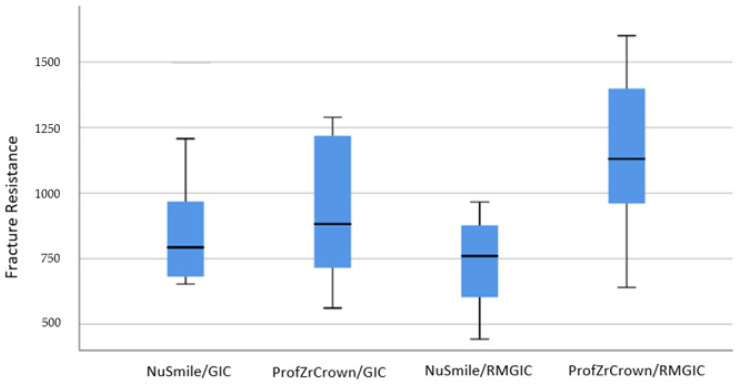
Box plot of the distribution of fracture strength measurements of prefabricated zirconium crowns.

The box plot of the distribution of fracture strengths obtained from zirconium crowns in the groups is shown in Figure 3.

### 3.2. Microleakage Test Results

**Figure 4:**
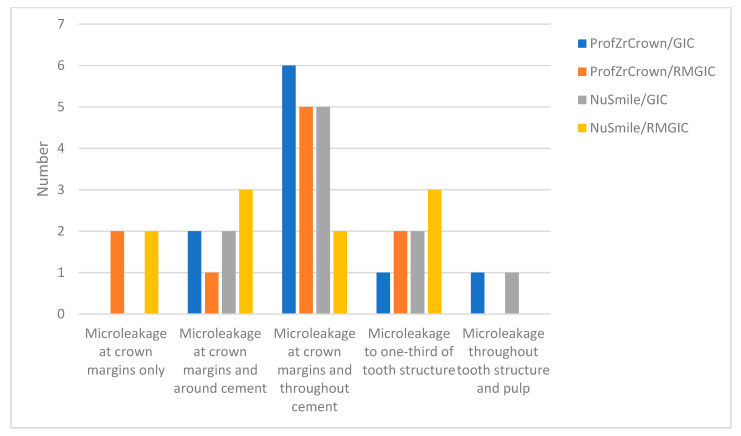
The distribution of microleakage values among the groups.

The microleakage values for each group are presented in Figure 4. Microleakage was lowest in ProfZrCrown/RMGIC. The highest microleakage scores were observed in NuSmile/GIC. Although variations in microleakage levels were observed across the groups, no statistically significant difference was found between them (*p* = 0.633, Fisher’s Exact Test).

## 4. Discussion

### 4.1. Clinical Requirements

In regions where oral hygiene and preventive dental care services are inadequate, primary teeth are prone to rapid caries progression, often resulting in significant loss of tooth structure [17]. In such cases, full-coverage restorations are considered a more appropriate treatment option, as they help preserve the tooth’s dimensional and structural integrity, thereby extending its functional lifespan. SSCs are widely used because of their durability; however, their poor aesthetics have driven the development of alternative materials [18]. PZCs, introduced in response to these limitations, offer superior aesthetic outcomes along with favorable mechanical and biological properties.

Parental preferences play a critical role in the selection of pediatric restorations. Studies have shown that parents often prioritize aesthetics over cost and durability [19]. As a result, the demand for tooth-colored crowns has increased for both anterior and posterior primary teeth.

Although long-term clinical data on PZCs remain limited, in vitro studies have demonstrated their high fracture resistance, flexural strength, and chemical durability [20]. In our study, the fracture strength and microleakage performance of a well-established zirconia crown system (NuSmile^®^) were compared with a newly introduced brand (ProfZrCrown^®^). Both crown types were cemented using two different glass ionomer-based luting agents and subjected to artificial aging protocols.

### 4.2. Study Protocol

In our study, natural primary teeth were used to more reliably ensure the modulus of elasticity and bonding properties between cement and natural teeth. Although variations in tooth size and anatomy limited full standardization, all preparations were carried out by a single operator to minimize procedural discrepancies. Natural anatomical variation is acknowledged as a limitation of this study.

The success of crown restorations depends on multiple factors, including the choice of material, the amount of tooth preparation, the type of cement used, the clinician’s skill, masticatory forces, cusp morphology, remaining tooth structure, and the adaptation of the gingival tissues [21].

For both crown types, the teeth were prepared according to the manufacturers’ recommendations with 1.5–2 mm occlusal reduction, 1 mm circumferential preparation, and approximately a 6° occlusal convergence angle [22]. No artificial periodontium simulation was performed; the teeth were embedded directly in acrylic resin. Although such a simulation may enhance clinical relevance, it can cause specimen mobility that potentially affects results [23].

### 4.3. Cement Selection

PZCs require greater tooth reduction, resulting in increased cement space, making the choice of cement a critical factor for restoration success [24,25]. Various luting agents can be used for cementation of zirconia crowns; however, there is no consensus on the ideal cement for primary teeth [26]. Both GIC and RMGIC have been shown to perform successfully in vivo and in vitro [27,28].

In a clinical study by Walia et al., all zirconia crowns cemented with glass ionomer cement (GIC) remained in place during a six-month follow-up period [10]. Similarly, Holsinger et al. reported that 83% of zirconia crowns cemented with GIC exhibited no debonding [29]. Raigrodski stated that zirconia oxide restorations do not require an adhesive interface for retention and can be conventionally cemented [30]. Quaas et al. also concluded that adhesive cementation is not necessary for zirconia crowns except in specific clinical conditions, such as short clinical crown height [31].

In a 36-month follow-up study by Azab et al., 50 primary first molars were restored using zirconia crowns cemented with either GIC or bioactive cement. Fewer debonded crowns were observed in the GIC group [32]. Similarly, studies by Mathew and Kist demonstrated that prefabricated zirconia crowns cemented with GIC yielded successful outcomes, with no significant difference in fracture resistance between GIC and bioactive cements [33,34]. In our study, prefabricated zirconia crowns were cemented using GIC (Ketac™ Cem Radiopaque) and RMGIC (Ketac™ Cem Plus). Following thermal cycling, no retention loss was observed in any group. Despite using different cement types, no statistically significant differences were found in fracture resistance or microleakage values. Based on these findings, it can be concluded that the type of cement does not have a significant effect on the fracture resistance of the crowns.

### 4.4. Thermal Cycle

Thermal cycling is widely used in in vitro studies to simulate aging and induce stress at the adhesive interface during microleakage and fracture resistance testing. The mismatch in the coefficients of thermal expansion between restorative materials and dental tissues can result in interfacial stresses and the formation of microcracks [23]. The effect of thermal cycling on marginal leakage is associated with the thermal conductivity and expansion characteristics of the luting agent used. Various thermal cycling protocols have been implemented to replicate intraoral temperature fluctuations. It has been reported that the average closed-mouth temperature is approximately 37 ± 1 °C, with maximum temperatures reaching 55 ± 1 °C or even 65 ± 1 °C, and minimum values dropping to as low as 4 ± 1 °C [35]. Palmer et al. recorded intraoral temperatures of up to 58.5 ± 3.3 °C in the maxillary anterior region and 53.1 ± 4.1 °C in the mandibular posterior region [36].

Addison et al. suggested that such thermal changes are short in duration, lasting about 5 s, and occur up to 10 times daily, noting that 3500 thermal cycles approximately simulate one year of clinical service [37]. Another study proposed that 10,000 cycles may represent the same clinical time frame [38].

Although there is no consensus in the literature regarding dwell time and number of cycles in thermal cycling protocols, shorter dwell times are thought to better mimic clinical conditions. In our study, to simulate intraoral conditions, 6000 cycles between 5 °C and 55 °C were applied, with a dwell time of 15 s in the water bath and 10 s in ambient air. However, thermal cycling cannot fully replicate intraoral conditions. Differences in humidity and temperature between laboratory and clinical settings may affect the cementation process, leading to variations in the initial setting reaction and final properties of the cement. This is considered one of the limitations of the present study.

### 4.5. Fracture Resistance

While normal masticatory forces are applied at an angle of 20–28° to the long axis of the teeth, in vitro studies generally apply these forces at a 90° angle [39]. In our study, specimens were also positioned perpendicular to the testing device, which can be considered a methodological limitation. According to the literature, the maximum bite force reported in children ranges from 106 to 433 N, whereas all samples in this study exhibited fracture resistance values exceeding these reported forces [40,41]. This finding suggests that both crown types possess sufficient durability for clinical use.

Şahin et al. evaluated the fracture resistance of zirconia crowns cemented with different luting agents. The highest fracture resistance was observed in crowns cemented with resin cement and GIC, while the lowest values were recorded with bioactive cement and RMGIC [42]. Similarly, in our study, NuSmile^®^ crowns cemented with GIC showed higher fracture resistance than those cemented with RMGIC. However, in the ProfZrCrown^®^ group, crowns cemented with RMGIC demonstrated greater fracture resistance.

In a randomized controlled trial conducted by Azab et al., 50 primary molars were restored with pediatric zirconia crowns using two different cementation materials (GIC and bioactive cement). No fractures were observed in any crowns regardless of the cement used, and high fracture resistance was reported after a 36-month follow-up, indicating that the cement type had no significant effect on clinical durability [32]. In an in vitro study by Abushanan et al., the fracture resistance of three different prefabricated zirconia crowns (NuSmile^®^, Cheng, and Spring EZ™) was investigated, with the highest resistance found in posterior Cheng zirconia crowns [43]. These variations may be attributed to differences in mold materials, crown composition and dimensions, type of luting agent used, and cement space thickness.

According to the fracture resistance results of our study, the ProfZrCrown^®^/RMGIC group showed the highest values, followed by ProfZrCrown^®^/GIC, NuSmile^®^/GIC, and the lowest in NuSmile^®^/RMGIC. These findings suggest that fracture resistance may be independent of the luting agent used. This can be attributed to their different compositions and microstructures, particularly with respect to yttria. A higher yttria content increases the translucency of zirconium but also results in lower fracture resistance [44,45]. Another key finding is that ProfZrCrown^®^ crowns, whether cemented with RMGIC or GIC, exhibited higher fracture resistance than NuSmile^®^ crowns. This difference is likely due to variations in material composition and crown dimensions. Based on these values, ProfZrCrown^®^ crowns demonstrate higher fracture resistance than the widely used NuSmile^®^ crowns and may serve as a promising alternative in clinical practice.

### 4.6. Microleakage

One of the clinical failure causes of prefabricated crowns is microleakage resulting from marginal gap formation [46]. Microleakage allows the passage of bacteria, fluids, and molecules at the tooth–restoration interface, potentially leading to discoloration, postoperative sensitivity, secondary caries, restoration failure, and pulpal pathology [23]. This condition generally arises from factors such as polymerization shrinkage, differences in thermal expansion coefficients between the restoration and tooth structure, microcracks, and inadequate surface treatment. Moreover, continuous exposure to saliva in the oral cavity may contribute to premature failure of the luting interface [47].

Although zirconia is a material with superior mechanical properties, conventional cementation techniques may not provide adequate bond strength. Yet, bond strength is critical for preventing microleakage and ensuring the long-term success of the restoration. Among the most commonly used methods for evaluating microleakage are dye penetration techniques with organic dyes. Due to its ease of use, low cost, and rapid results, 2% basic fuchsin solution is frequently preferred. However, the two-dimensional nature of this method and its limited reflection of clinical conditions are significant limitations [48]. Therefore, more comprehensive methods, such as micro-CT, scanning electron microscopy (SEM), and bacterial penetration, can be used in future studies.

In our study, two different brands of prefabricated zirconia crowns (NuSmile^®^ and ProfZrCrown^®^) were cemented using a glass ionomer cement (GIC; Ketac™ Cem Radiopaque) and a resin-modified glass ionomer cement (RMGIC; Ketac™ Cem Plus). Microleakage levels were evaluated using a 2% basic fuchsin solution. The specimens were immersed in the dye solution for 24 h and then rinsed under running water, sectioned, and examined under a stereomicroscope at 30× magnification. The scoring system used by Stepp et al. was employed for microleakage assessment [27].

In the study by Stepp et al., 40 extracted permanent molars were restored using two brands of prefabricated zirconia crowns (Ez-Pedo™ and NuSmile^®^), GIC (Ketac™ Cem), and a bioactive cement (Biocem). Microleakage was observed in all groups. NuSmile^®^ crowns cemented with Biocem exhibited lower microleakage than those cemented with GIC. Additionally, the NuSmile^®^/Biocem combination showed less leakage compared to the Ez-Pedo™/GIC combination [27].

Similarly, a study involving extracted primary molars restored with prefabricated zirconia crowns using GIC and RMGIC reported statistically comparable microleakage values for both cement types [48]. Consistent with these findings, our study revealed no statistically significant difference in microleakage between the GIC and RMGIC groups, with the majority of observed leakage scores falling within grades I, II, and III. This outcome may be attributed to the use of non-vital extracted teeth, as the absence of dentinal fluid flow eliminates the natural barrier against bacterial penetration observed in vital teeth.

Our study also compared the microleakage levels of two different prefabricated zirconia crown brands cemented with different luting materials. Although no statistically significant differences were detected among the groups, Ketac™ Cem Plus yielded slightly lower microleakage scores compared to Ketac™ Cem Radiopaque.

Since zirconium does not exhibit sufficient bond affinity to luting cement, surface treatments are thought to play an important role in creating a strong bond between zirconium restorations and tooth structure [49]. Micromechanical treatments such as sandblasting and laser etching, as well as the use of primers containing 10-MDP, enhance the adhesion properties of zirconia and strengthen bond strength [50,51]. Although appropriate surface treatments and luting cement selection are considered critical for the success of PZCs in pediatric dentistry, the most effective methods have not yet been clarified [52]. This necessitates further investigation of the effects of different surface treatments and resin cements on the bond strength and clinical success of PZCs.

In conclusion, both crown and cement combinations used in this study demonstrated fracture resistance values that far exceed the average bite forces reported in children and showed clinically acceptable microleakage levels. ProfZrCrown^®^, a newly manufactured zirconia crown brand with no prior studies in the literature, exhibited higher fracture resistance than NuSmile^®^ crowns with both types of cement while performing similarly in terms of microleakage. These findings suggest that ProfZrCrown^®^ may represent a viable alternative among prefabricated zirconia crowns. This study may serve as a foundation for future comprehensive in vivo and in vitro research on this newly developed material.

## 5. Conclusions

Within the limitations of this in vitro study, both zirconia crown brands and the luting cements used demonstrated clinically acceptable fracture resistance and microleakage values following thermocycling equivalent to one year of aging. No crown debonding was observed in any of the groups. Overall, ProfZrCrown^®^ crowns exhibited higher fracture resistance regardless of the type of cement used. Microleakage analysis revealed no statistically significant differences among the groups (*p* > 0.05); however, crowns cemented with RMGIC showed lower microleakage compared to those cemented with GIC. Based on these findings, the newly developed ProfZrCrown^®^ crowns may be considered a durable and aesthetic alternative for the restoration of primary molars with extensive structural loss. Nevertheless, further advanced in vivo and in vitro studies are necessary to support these results and evaluate long-term clinical performance.

## Figures and Tables

**Table 1:** Composition and manufacturers of the crown materials used in this study.

Crown Brand	Chemical Composition	Manufacturer
ProfZrCrown^®^	ZrO_2_ 71.9% Y_2_O_3_ 5.6% HfO_2_ 12.9% Al_2_O_3_ < 0.05%, other oxides < 1%	ProfZrCrown, Istanbul, Turkey
NuSmile^®^	ZrO_2_ 88–96% Y_2_O_3_ 4–6% HfO_2_ 5%	NuSmile, Houston, TX, USA

**Table 2:** Category of microleakage scores.

Category	Description
0	Microleakage at crown margins only
1	Microleakage at crown margins and around cement
2	Microleakage at crown margins and throughout cement
3	Microleakage to 1/3 of tooth structure
4	Microleakage throughout tooth structure and pulp

**Table 3:** Comparison of the fracture resistance (N) of prefabricated zirconia crowns.

Crown Type	Luting Cement	n	Mean ± SD	Min	Max	*p* †
NuSmile	GIC	10	884.58 ± 263.65 ^a^	653.11	1452.00	**0.021**
	RMGIC	10	736.05 ± 165.12 ^b^	561.68	966.09	
ProfZrCrown	GIC	10	947.50 ± 274.65 ^c^	443.03	1288.96	
	RMGIC	10	1145.58 ± 306.56 ^a^	640.17	1599.93	

†: Kruskal–Wallis. There is a significant difference between the same superscripts in the table (*p* < 0.05).

## Data Availability

The original contributions presented in this study are included in this article. Further inquiries can be directed to the corresponding authors.

## Abbreviations

The following abbreviations are used in this manuscript:

AAPD	American Academy of Pediatric Dentistry
SSCs	Stainless steel crowns
SCs	Resin composite strip crowns
PVSSCs	Rre veneered stainless steel crowns
PZCs	Prefabricated zirconia crowns
GIC	Glass ionomer cement
RMGIC	Resin-modified glass ionomer cement
